# There is no difference in contagious yawning between men and women

**DOI:** 10.1098/rsos.160174

**Published:** 2016-09-07

**Authors:** Andrew C. Gallup, Jorg J. M. Massen

**Affiliations:** 1 Department of Psychology, State University of New York at Oneonta, Oneonta, NY, USA; 2 Department of Cognitive Biology, University of Vienna, Vienna, Austria

Norscia *et al.* [1] recently reported the first evidence for a sex bias in contagious yawning among humans. Based on previous research showing an indirect connection between contagious yawning and empathy (e.g. [2–4], but see [5,6]) and that levels of empathy appear to be higher in women compared with men (e.g. [7–9]), the authors investigated whether there is also a sex difference in the expression of contagious yawning through an observational study on humans. By examining 92 dyads already showing evidence of contagious yawning, which was a subset of a much larger sample of individuals, Norscia *et al.* report that both social bond and sex were significant predictors of this response. In particular, contagious yawning was more common if the dyad was strongly bonded and when the individual catching the yawn was female.

Here, we call into question these conclusions about a sex difference in contagious yawning. First, we draw attention to more than a dozen previous publications on contagious yawning in humans, many of which were not cited by Norscia *et al.* showing no difference in susceptibility to yawn contagiously and/or contagious yawning frequency between men and women. Similarly, we do the same for nearly a dozen unacknowledged publications in the comparative literature. Lastly, we challenge the validity of the reported sex difference within the Norscia *et al.* study itself, based on their analysis of only a restricted subset of documented contagious yawners.

A close examination of the empirical literature clearly shows that the sex difference reported by Norscia *et al.* stands in stark contrast to previous findings on contagious yawning in humans. While Norscia *et al.* only reference a single publication that did not find a difference in the frequency of contagious yawning between men and women [5], we provide references to an additional 14 papers showing no effect (two of which were in fact written by some of the Norscia *et al.* authors in question; table 1). Furthermore, within the 15 total publications showing no effect, two include two independent studies and samples. Therefore of the 16 papers (18 samples) that have addressed the question of a sex difference in contagious yawning, only Norscia *et al.* found a statistically significant effect between men and women.

**Table 1.** Publications other than Norscia *et al.* [1] that test for a sex difference in contagious yawning.

publication (chronological)	sample *n* (F)	setting	stimulus	measure	contagion variable	sex bias
Platek *et al.* [2]	*n* = 65 (34)	laboratory	video clips	objective	continuous	no effect
Gallup & Gallup [10]	*n* = 44 (27)	laboratory	video clips	objective	binary and continuous	no effect
(two studies)	*n* = 33 (20)					
Senju *et al.* [11]	*n* = 49 (15)	laboratory	video clips	objective	continuous	no effect
Senju *et al.* [12]	*n* = 62 (23)	laboratory	video clips	objective	continuous	no effect
Helt *et al.* [13]	*n* = 123 (78)	laboratory	video clips	objective	binary	no effect
Gallup & Eldakar [14]	*n* = 160 (90)	naturalistic	static images	self-report	binary	no effect
Norscia & Palagi [15]	*n* = 109 (56)	naturalistic	live target	objective	binary and continuous	no effect
Usui *et al.* [16]	*n* = 72 (27)	laboratory	video clips	objective	binary and continuous	no effect
(two studies)	*n* = 51 (19)					
Bartholomew & Cirulli [5]	*n* = 328 (220)	laboratory	video clips	self-report	binary and continuous	no effect
Massen *et al.* [17]	*n* = 120 (72)	naturalistic	static images	self-report	binary and continuous	no effect
Palagi *et al.* [18]	*n* = 44^a^	naturalistic	video clips	objective	continuous	no effect
Eldakar *et al.* [19]	*n* = 142 (70)	naturalistic	static images	self-report	binary	no effect
Massen *et al.* [20]	*n* = 118 (82)	laboratory	static images	self-report (and objective)^b^	binary	no effect
Rundle *et al.* [21]	*n* = 135 (78)	laboratory	video clips	objective	binary and continuous	no effect
Gallup *et al.* [22]^c^	*n* = 105 (79)	laboratory	video clips	self-report	binary and continuous	no effect

^a^Palagi *et al.* [18] fails to indicate the final sample of men and women represented in the 22 dyads observed in the study.

^b^A subset of this sample (10 males and 12 females in yawning condition) was video-recorded and coded objectively afterwards. Also here, we found no sex difference in the susceptibility to contagiously yawn between males and females (Fisher exact: *p* = 0.204).

^c^This is the only study that was published after Norscia *et al.* [1].

Given the logic of statistical probability, the most parsimonious explanation for this pattern of results is that the null effect is real and the sex difference reported by Norscia *et al.* represents a false positive. In fact, if the false positive rate is 5% (*p* = 0.05), then the cumulative percentage chance of discovering less than or equal to 1 significant effects out of the 18 total samples is greater than 77% (binomial test). The odds of finding one or more significant effects are greater than 60%. If the recently reported sex bias is indeed real, however, the chance of 17 independent samples showing no effect is highly improbable. Even if there was an equal likelihood of expecting a sex bias or a null effect (much like flipping a coin), which would be highly conservative, the odds of discovering 17 null effects out of 18 samples is less than 0.01% (*p* = 0.00007). Although a variety of methods and measurements were used across these studies for both eliciting and measuring contagious yawning (table 1), there is no *a priori* reason to believe this would alter the expression of yawns in men versus women consistently in one direction or the other across these samples. In fact, these differences in methodology across studies suggest that the failure to find a sex difference is a robust and easily replicable effect.

Norscia *et al.* draw on comparative/animal literature to support the sex difference they found in humans, by citing other non-human examples in which there is a ‘female skew’ for contagious yawning. However, just as in the case of their examination of the human literature, there are papers that Norscia *et al.* do not cite in which no effect of sex is reported (one of which was written by the authors in question). For example, no main effect of sex has been observed for chimpanzees [4,23–25], bonobos [18], domesticated dogs [26–29] and budgerigars [30]. There is also no sex difference for video-induced yawning in stump-tailed macaques, although it is not clear whether this response represents a mechanism of contagion [31]. Moreover, the findings supporting a female bias in non-humans do not actually describe a female bias that is comparable to what Norscia *et al.* report for humans; i.e. a main effect of the sex of the receiver on the frequency of contagious yawning. For example, female wolves show a shorter reaction time to yawn than males but do not differ in frequency [32], bonobos are more likely to yawn to a female model but the sex of the receiver is not a significant predictor [33], and for gelada baboons female–female dyads showed more contagious yawning than female–male but no comparison is made for this response in male–male pairs [2]. Arguing along those lines, Massen *et al.* [24] actually found opposing results in chimpanzees, whereby male yawns were more contagious than female yawns and male–male dyads showed most contagious yawning. Whereas there have been interesting hypotheses posed to explain the differences in yawn contagion between species with regard to the compositions of the trigger and responder, it is interesting that Norscia *et al.* do not test for this dyadic aspect (i.e. the interaction effect of the trigger's and receiver's sex) in their study. What we can conclude, however, is that the effects described in the comparative literature are far more mixed than what Norscia *et al.* originally reported.

Lastly, we question the validity of the conclusions derived by Norscia *et al.* about a sex bias in yawn contagion among humans. The analytic strategy they employed did not actually assess whether there was a difference in susceptibility to yawn contagiously between men and women, and consequently also did not assess whether there was a difference in contagious yawning frequency in the total sample of men and women in their study. Instead, the authors used a particular set of exclusion criteria and only analysed data from dyads in which (i) yawn contagion was present and (ii) at least three independent occasions of contagious yawning were available. Although the authors rightfully argue that this ensures that yawn contagion is correctly detected, these methods omit relevant zeros, especially when taking into account that the number of occasions to show yawn contagion varied across individuals. In general (table 1), contagious yawning is at first analysed by assessing what affects whether an individual will or will not yawn (0/1) after witnessing a yawn. In fact, the authors reported in a previous paper [15], on a subsample of the study in question, that no sex difference was found regarding the probability to yawn contagiously. In addition, the authors did not assess contagious yawning among strangers. All these (and additional) decisions restricted the analysed sample to 34.5% of the total dataset. What Norscia *et al.* can conclude from their analysis is that of the men and women that met these criteria for contagious yawning, females yawned more than men in response to yawning stimuli. They do not show that females are more susceptible to contagious yawning, but only that among those who are susceptible within their restricted sample women are more likely to do so. Thus, the results reported by Norscia *et al.* are inconclusive if not misleading.

In summary, the available evidence on contagious yawning in humans shows that there is no difference between men and women. We have identified a total of 15 other publications (consisting of 17 study samples) that have addressed this question, and all report no effect of sex in terms of the susceptibility to contagious yawning and/or the frequency of contagious yawning. Furthermore, we have provided additional evidence for a much more mixed picture for the comparative literature on this topic. Therefore, until the sex difference reported by Norscia *et al.* is replicated independently by other investigators, it should be considered a false positive. Furthermore, the conclusions reached by Norscia *et al.* are misleading in that they imply a global effect of sex on the susceptibility to yawn contagiously, when in fact their analyses were restricted to a subset of their sample that already showed abundant evidence of contagious yawning. As for contagious yawning as an indirect marker for empathic processing, we are left to conclude that the consistent lack of a female bias in the literature fails to support this connection.
